# Spatiotemporal Gradients of PAH Concentrations in Greek Cities and Associated Exposure Impacts

**DOI:** 10.3390/toxics12040293

**Published:** 2024-04-16

**Authors:** Irini Tsiodra, Kalliopi Tavernaraki, Georgios Grivas, Constantine Parinos, Kyriaki Papoutsidaki, Despina Paraskevopoulou, Eleni Liakakou, Alexandra Gogou, Aikaterini Bougiatioti, Evangelos Gerasopoulos, Maria Kanakidou, Nikolaos Mihalopoulos

**Affiliations:** 1 Institute for Environmental Research and Sustainable Development, National Observatory of Athens, 15236 Athens, Greece; itsiodra@noa.gr (I.T.); popi_tavernaraki@yahoo.gr (K.T.); ggrivas@noa.gr (G.G.); dparask@noa.gr (D.P.); liakakou@noa.gr (E.L.); egera@noa.gr (E.G.); nmihalo@noa.gr (N.M.); 2 Environmental Chemical Processes Laboratory, Department of Chemistry, University of Crete, 71003 Heraklion, Greece; kyriakipapou@gmail.com (K.P.); mariak@uoc.gr (M.K.); 3 Institute of Oceanography, Hellenic Centre for Marine Research, 19013 Anavyssos, Greece; ksparinos@hcmr.gr (C.P.); agogou@hcmr.gr (A.G.); 4 Center for Studies of Air Quality and Climate Change, Institute for Chemical Engineering Sciences, Foundation for Research and Technology Hellas, 26504 Patras, Greece; 5 Institute of Environmental Physics, University of Bremen, 28359 Bremen, Germany

**Keywords:** polycyclic aromatic hydrocarbons, benzo(a)pyrene, diagnostic ratio, biomass burning, exposure risk, Greece, Eastern Mediterranean

## Abstract

To study the spatiotemporal variability of particle-bound polycyclic aromatic hydrocarbons (PAHs) and assess their carcinogenic potential in six contrasting urban environments in Greece, a total of 305 filter samples were collected and analyzed. Sampling sites included a variety of urban background, traffic (Athens, Ioannina and Heraklion), rural (Xanthi) and near-port locations (Piraeus and Volos). When considering the sum of 16 U.S. EPA priority PAHs, as well as that of the six EU-proposed members, average concentrations observed across locations during summer varied moderately (0.4–2.2 ng m^−3^) and independently of the population of each site, with the highest values observed in the areas of Piraeus and Volos that are affected by port and industrial activities. Winter levels were significantly higher and more spatially variable compared to summer, with the seasonal enhancement ranging from 7 times in Piraeus to 98 times in Ioannina, indicating the large impact of PAH emissions from residential wood burning. Regarding benzo(a)pyrene (BaP), an IARC Group 1 carcinogen and the only EU-regulated PAH, the winter/summer ratios were 24–33 in Athens, Volos, Heraklion and Xanthi; 60 in Piraeus; and 480 in Ioannina, which is afflicted by severe wood-burning pollution events. An excellent correlation was observed between organic carbon (OC) and benzo(a)pyrene (BaP) during the cold period at all urban sites (r^2^ > 0.8) with stable BaP/OC slopes (0.09–0.14 × 10^−3^), highlighting the potential use of OC as a proxy for the estimation of BaP in winter conditions. The identified spatiotemporal contrasts, which were explored for the first time for PAHs at such a scale in the Eastern Mediterranean, provide important insights into sources and controlling atmospheric conditions and reveal large deviations in exposure risks among cities that raise the issue of environmental injustice on a national level.

## 1. Introduction

Polycyclic aromatic hydrocarbons (PAHs) are toxic organic pollutants that have the potential to induce carcinogenic effects following chronic inhalation exposure [1,2]. PAHs in the atmosphere can be present either in the gaseous or in the particulate phase, and although semi-volatile PAHs have the potential to initiate genotoxic mechanisms at the cellular level, heavier and less volatile PAH members with more rings and bay- or fjord-like regions [3,4] in their structure are more susceptible to enzymatic metabolism, leading to DNA adduction and mutations. Besides their carcinogenic activity [5], PAHs are also related to a plethora of non-carcinogenic—e.g., cardiorespiratory—effects, which are mainly induced by oxidative stress and inflammatory responses [6,7]. Moreover, PAHs have been linked to neurodevelopmental effects following prenatal or early postnatal exposure, even at lower doses than those that induce carcinogenicity [8,9,10]. Regarding individual PAHs, naphthalene, benzo(a)anthracene, chrysene, benzo(b)fluoranthene, benzo(k)fluoranthene, Indeno(123cd)pyrene and dibenz(a,h)anthracene are classified as Group 1, 2A or 2B carcinogens by IARC (IARC Monographs 2010). Moreover, members like phenanthrene and benzo(ghi)perylene have been linked to cardiovascular and other systemic effects [7,11,12].

In 1976, the United States Environmental Protection Agency (U.S. EPA) established a list of 16 harmful PAH members that should be monitored regularly [13]. The European Union also moved to regulate atmospheric PAHs in 2004 (directive 2004/107/EC), setting an annual target value only for BaP (1 ng m^−3^) [14]. However, in the directive, it was proposed that PAH measurements should not be limited only to BaP but should also include benzo(a)anthracene, benzo(b)fluoranthene, benzo(j)fluoranthene, benzo(k)fluoranthene, indeno(1,2,3-cd)pyrene and dibenz(a,h)anthracene.

Fine particle-bound PAHs originate from a variety of anthropogenic activities, mostly related to incomplete combustion of fossil fuels and biomass [15,16], while fugitive emissions from fuel oil transport, use, storage and maturation can also be a factor in specific environments. These source categories can be classified as pyrogenic and petrogenic, respectively [17].

Recent studies have highlighted the connection of particle PAHs with residential biomass burning and have reported this source as abundantly present in European cities, where it frequently leads to the formation of winter haze events [18]. The use of biomass as a heating fuel alternative has increased dramatically in the last decade following the 2008 global recession and has persisted throughout the ongoing energy crisis, leading to an alarming increase of the fine particle carcinogenic potential [19,20].

In Greece, over the last few decades, research on PAHs and their sources has mostly been conducted through single-site measurements [18,21,22], hindering the study of contrasts between different site types and at the spatial scale (within-area or between-area), which has crucial implications for exposure assessment and also for informed policymaking. At present, spatial data regarding exposure to PAHs have to be mined from infrequent, fragmented and asynchronous observations. Furthermore, official PAH measurements according to the provisions of the EU directive in Greece are limited to a couple of monitoring sites in the Greater Athens Area and are reported only for BaP. Therefore, there is an urgent need for the longitudinal characterization of PAH levels in Greek cities, especially given the gravity of the residential wood-burning issue [18,23,24]. Moreover, measurements at different site types are needed to highlight the relative importance of residential against road and maritime transport sources.

Greece has been regularly reporting compliance with the BaP EU standard; however, this is assessed through measurements only at urban background sites around Athens, and it is unknown whether BaP annual mean concentrations remain- below the EU target value at traffic locations or in residential areas in cities with increased heating energy demand during the cold season. In this context, we investigated the seasonal pattern of PAH concentrations in six cities (Athens, Piraeus, Ioannina, Volos, Xanthi, Heraklion), using measurements at varying site types (traffic, urban, rural, port) [25].

The majority of these cities were previously characterized in terms of PM_2.5_ levels, sources and long-term variability in an overview study [26], which reported low and almost uniform PM_2.5_ levels over continental Greece during the warm period. Conversely, during the cold period, PM_2.5_ concentrations were largely increased in all areas, due to residential biomass burning. Specifically for the study sites, in Athens, the National Observatory of Athens (NOA) Thissio supersite has been well-characterized during the last decade in terms of toxic organic aerosol sources and chemical composition [18,23,27,28]. Ioannina is a medium-sized mountainous city impacted by intense residential biomass burning (BB), especially during winter nights. These emissions, in combination with the city’s climate and topography (mountainous basin, air mass stagnation, haze-promoting conditions), result in a large accumulation of organic aerosols, which is also due to local-scale secondary processing [29,30]. Organic aerosol sources were also recently characterized at the port of Piraeus [31]. Little is known about the organic aerosol composition in medium-sized peripheral Greek cities like Volos, Heraklion and Xanthi [26], although some results on PM concentration levels have been reported. Frequent exceedances of the PM_10_ daily limit values have been consistently reported in Volos [32]. In Heraklion, the frequency of exceedance of the PM_10_ limit value in summer and fall can be almost double compared to the other half of the year, despite a large number of spring exceedances due to dust transport [33]. In the near-city rural site of Xanthi, the aforementioned overview study indicated summer PM_2.5_ levels comparable to those measured in other urban background sites in Greece, while in winter, the mean concentration was 70% lower compared to the most polluted city (Ioannina) [26].

In this work, focus was placed on the specific PAH congeners regulated by the EPA and the EU. The selected measurement areas had different characteristics and populations, allowing to assess the impact of PAH sources and evaluate potential exposure risks on a national level. Τhis was performed for the first time at multiple sites across Greece, highlighting the importance of representative PAH measurements for a deeper understanding of their origin and consequently of mitigation possibilities. A key motivation of this work was to examine whether a potentially severe ambient PAH pollution issue in Greece is masked by the lenient macro-siting requirements foreseen in current environmental legislation. To our knowledge, this study is one of the few worldwide [34,35,36,37] to investigate the PAH spatial variability in national networks on a seasonal basis, while comparing different measurement area types (urban, rural) and site types (traffic, near-port, background). This comparison is expected to highlight environmental injustices regarding carcinogenic ambient exposure among areas that experience different climate but also socio-economic characteristics, especially when the option to use “clean” residential heating fuels is not available.

## 2. Materials and Methods

### 2.1. Study Area and Filter Sampling

**Figure 1:**
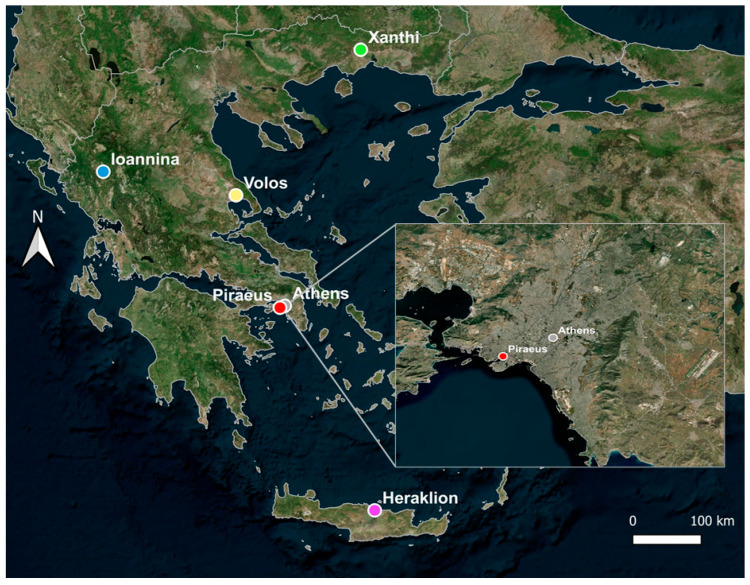
Map of Greece displaying the six cities where PAH measurements were conducted. The two sites in the Greater Area of Athens are shown in the insert panel.

Measurements were conducted at sites in six Greek cities (Figure 1), namely Athens (3.5 M inhabitants, urban background site), Heraklion (0.2 M, traffic site), Ioannina (0.15 Μ, urban background site), Xanthi (0.05 M, rural background site), Piraeus (0.6 M, urban site near the port) and Volos (0.15 M, urban site near the port) (Figure 1, Table 1). Site characterizations are according to the 2008/50/EC directive and European Environmental Agency (EEA) guidelines [38].

Ambient particle samples were collected on quartz-fiber filters (Flex Tissuquartz, Pall Corporation, Port Washington, NY, USA) using high- or low-volume samplers, during winter (December and January) and summer (June to August) months. All used gravimetric samplers were reference-equivalent for PM measurement according to the EU standards. The sampling duration at all sites was 24 h. Sampling took place concurrently at Ioannina, Xanthi, Volos and Piraeus [31] in summer (June to August 2019) and winter (December 2019 to January 2020). In Athens, the same monthly periods were considered, but for summer 2017 and winter 2017–2018 instead [18]. In Heraklion, the same monthly periods were considered for summer 2022 and winter 2022–2023. This was necessary due to the lack of concurrent measurements with the other four sites and to ensure a degree of seasonal comparability. It is noted that a lack of significant interannual trend for black carbon (a pollutant strongly associated with primary PAHs) was reported for year 2015–2019 at the Athens Thissio site [39]. Moreover, the selected periods in Heraklion presented very similar meteorological conditions (temperature, wind speed) to the respective months in 2019–2020.

PM_2.5_ particles were collected at all sites, with the exception of Heraklion where a PM_10_ sampler was used, since the station is operated officially according to the provisions of the 2004/107/EC directive. Nevertheless, this is not anticipated to significantly influence the comparison with PAH concentrations measured in PM_2.5_, since the vast majority (85–95%) of PAH species determined in this study are expected to be present in the PM_2.5_ fraction [40,41,42,43,44,45,46,47]. Past measurements in Heraklion showed that 97% and 93% of total particle-bound Σ-PAH concentrations were encountered incomposed of particles smaller than 3 μm and 1.5 μm, respectively [48]. Similar results have been reported in Athens, with 98–99% of PM_10_ Σ-PAHs measured in the PM_2.5_ fraction [47]. The dominance of the fine mode in Σ-PAH concentrations has also been demonstrated in Thessaloniki, where 88–89% of mean ambient Σ-PAHs concentrations were attributed to particles up to 3 μm [44].

Filters were equilibrated pre-/post-sampling, and weighed under controlled temperature (20 ± 3 °C) and relative humidity (40 ± 5%) and were stored in a freezer until analysis. Field blanks and laboratory blanks were also kept and analyzed. A total of 305 samples were collected and analyzed (81 in Ioannina, 71 in Thissio, 62 in Xanthi, 38 in Piraeus, 29 in Volos, 23 in Heraklion). Details regarding measurements and meteorological data at the six sites are provided in Table 1 and Table 2.

### 2.2. Laboratory Analyses

PAHs in the filter samples were quantified by gas chromatography–mass spectrometry (GC-MS). An Agilent 6890N system (Agilent Technologies Inc., Santa Clara, CA, USA) with a J&W DB-5MS capillary column and a 5973 mass selective detector was used for samples from Athens, Piraeus, Volos and Xanthi. For samples from Ioannina and Heraklion, an Agilent 7890 (Agilent Technologies Inc., Santa Clara, CA, USA) system with an HP-5MS capillary column and an Agilent 5975C mass selective detector was used [18,49]. All quartz filter samples were extracted using an accelerated solvent extraction system (Dionex ASE 300; Thermo Fisher Scientific Inc., Waltham, MA, USA). Prior to the analysis, samples were spiked with a mixture of deuterated internal standards for the identification of PAHs and calculation of recovery efficiencies (16 members, LGC Standards, Middlesex, UK). For the extraction, part of the quartz filter was extracted using a 50:50 n-hexane-dichloromethane (SupraSolv^®^, Merck KGaA, Darmstadt, Germany) mixture, and obtained extracts were purified through a silica column. PAHs were eluted with a 10 mL n-hexane/ethyl acetate (9:1 *v*/*v*) mixture and placed into a glass vial for further concentration under a gentle nitrogen stream [18,50,51]. [^2^H_12_]perylene (LGC Standards, Middlesex, UK) used as an internal standard for the calculation of recovery efficiencies, was spiked into the vial before sealing and storage. On the day of the analysis, injections with internal standards were also run to calculate Relative Response Factors (RRF).

The identification of compounds was based on the retention time, mass fractionation and co-injection of standard mixtures. Information regarding the detected PAHs is provided in Table 3. PAH quantification was based on the calculation of RRF for every PAH member. The recovery efficiency of the method was calculated using 17 deuterated PAHs (16 deuterated EPA PAHs and [^2^H_12_]perylene), according to the equation provided by Mandalakis et al., 2001 [52]. The calculated average recoveries for the two GC-MS systems, Agilent 6890N and Agilent 7890, were 81 ± 10% and 75 ± 12%, respectively, in agreement with literature values [50,53].

Limits of detection (LODs) were calculated as three times the standard deviation of blanks. All 16 EPA species were detected in blanks. For the summer period, mean blank concentrations were one order of magnitude lower than the mean measured concentrations, while during winter, they were almost two orders of magnitude lower. All data were corrected using the mean concentration of the blank samples, and correction for recoveries was also performed.

Organic and elemental carbon (OC, EC) concentrations were also determined by-the thermal–optical transmission (TOT) method, using a sunset carbon analyzer (Sunset Laboratory Inc., Portland, OR, USA) [54,55]. The LODs for OC and EC were estimated at 0.62 and 0.05 μg C m^−3^, respectively, with uncertainties of less than 10% and the final concentrations being blank corrected.

Meteorological data (temperature, relative humidity, wind speed and direction) were measured in situ at the Athens and Ioannina sites and were retrieved by the nearest stations of the automatic weather station network of the National Observatory of Athens (NOA) at the other four sites [56].

### 2.3. Carcinogenic Risk

It is well established that PAHs are strongly associated with increased carcinogenic risks. The carcinogenic risk from cumulative exposure to PAHs can be assessed by using the “toxicity equivalent factor” (TEF) approach, where the BaP equivalent concentration of each toxic PAH member is calculated based on its concentration and specific TEF conversion factors (Table 3) derived from toxicological studies [57,58]. The BaP-equivalent concentration (BaP_eq_) for the mixture of the 16 EPA PAHs (Σ_16_PAHs) was calculated according to the following equation:

(1) $${\Sigma BaP}_{eq}=\Sigma (C_{i}\;{TEF}_{i})$$

where C_i_ is the concentration (in ng m^−3^) and TEF_i_ is the toxicity equivalent factor for each member [57,59].

Having calculated the BaP_eq_, it is possible to estimate the incremental lifetime excess cancer risk (ILCR) from inhalation, based on the following equation:

(2) $$ILCR=\Sigma {BaP}_{eq}{UR}_{BaP}$$

where *UR_BaP_* (unit risk) is an estimation of the increased cancer risk from inhalation exposure to a concentration of 1 ng m^−3^ of BaP over a lifetime of 70 years. For this estimation, two approaches are commonly used. The more conservative is the one proposed by the Office of Environmental Health Hazards Assessment (OEHHA) of the California Environmental Protection Agency (CalEPA), with an inhalation UR_BaP_ (IUR) of 1.1 × 10^−6^ (i.e., 0.11 cases per 100,000). The other has been suggested by the Word Health Organization (WHO), with an IUR equal to 8.7 × 10^−5^ (i.e., 8.7 cases per 100,000) [18,53,60].

## 3. Results

### 3.1. Characterization of PAH Levels

Table 4 presents the winter–summer mean concentrations of the 16 EPA PAH sum (Σ_16_PAHs) along with BaP at the six cities. Considering the average of winter and summer values as an approximation of the annual mean, the value in Ioannina stands out (2.4 ng m^−3^), being well above the EU annual target value of 1 ng m^−3^. The Σ_16_PAHs sum in Ioannina was more than two times higher compared to the second-highest measuring site (Athens). The only study that had reported PAH concentrations in Ioannina [61] was based on measurements in the late 90s, and presented annual BaP concentrations varying between 0.3 and 2.6 ng m^−3^. However, these high levels had been attributed to excessive emissions from old-technology cars in Greece (pre-Euro 2, most of which were not even equipped with catalytic converters) and to heating oil burning. This illustrates a long-term organic air pollution issue for the city, albeit due to changing environmental pressures. PAH levels of the magnitude observed in Ioannina are rarely observed in European cities, except in areas directly impacted by fossil fuel-burning for power production, like in Upper Silesia, Poland [62]. However, they are more comparable to concentrations reported in East Asian cities [63,64,65,66].

At the Thissio urban background site in Athens, the annual approximation for BaP (0.76 ng m^−3^) was close to the EU target value of 1 ng m^−3^ (2004/107/EC directive). These levels are much higher than those measured (0.01–0.10 ng m^−3^) at the suburban background sites used in Greek reporting- to the EU. The concentrations observed at Thissio classify Athens among the upper tier of major European metropolitan areas reporting BaP concentrations at urban background sites [67]. BaP and Σ_16_PAH levels in winter appear similar to those recorded during the cold period in Southeastern-European capitals like Sofia [68], Zagreb [69], and Ljubljana [70].

At the two near-port sites (Piraeus and Volos), the estimated annual BaP concentration was also close to the EU target value of 1 ng m^−3^ (0.61 and 0.74 ng m^−3^, respectively), probably due to the proximity to shipping emissions and the increased traffic-related activity. Piraeus is the biggest port in Greece with more than 5.8 M passengers (at the central passenger terminal, within 200 m of the sampling site) and 600,000 tons of cargo annually [71]. In comparison to Piraeus, the port of Volos is much smaller, and its operation is concentrated on freight rather than on passenger transport. Similar annual BaP levels (0.60 ng m^−3^) were registered in Volos in 2015 [72]. Up to now, there has been no published record of PAH measurements in the port of Piraeus, but the estimated annual value for Σ_16_PAHs is close to the one reported in 2018–2019 (9.8 ng m^−3^) in the industrialized coastal area of the Thriassion plain (12 km to the NW of Piraeus), which is also affected by shipping emissions [73]. The summertime Σ_16_PAH levels in the two port areas (Piraeus and Volos) resemble those reported in other ports in the Mediterranean, like Venice [74], Brindisi [75], Bari and Taranto [76].

Mean annual estimates in Heraklion were 60% lower than the respective ones in Piraeus, in spite of the site’s traffic characterization. This can be attributed to the low winter levels due to the mild temperature conditions [77] and reduced heating needs. However, the traffic influence can be seen by comparison to the 2-year average background levels (0.08 ng m^−3^) outside the city, measured in 2012–2014 [78]. Heraklion is the only city in the study where vehicular traffic remains stable if not increased during the summer period due to the tourist activity (indicatively there were 1.9 million arrivals at the Heraklion international airport in July–August 2022) [79]. However, it should be noted that current information about the impact of direct road traffic on PAH levels in Greece is very limited, since the latest systematic PAH measurements at traffic sites were conducted more than 10 years in the past [42,80,81], and in the years between, there have been significant changes in the road transport sector, including the modernization passenger fleet and the increased participation of diesel cars.

The rural background site in the area of Xanthi expectedly registered the lowest winter–summer mean (0.17 ng m^−3^), given the relative absence of local sources. However, it is noteworthy that this value is considerably higher than the one (0.06 ng m^−3^) reported by 2-year measurements at a similar site in the same region of Greece (Thrace), near the city of Alexandroupolis, during 2009–2011 [82], i.e., prior to the onset of the Greek recession. This might signify that even the near-city background locations in Greece can be affected by the dramatically increased winter residential wood burning (RWB) emissions in the past decade. A similar impact of RWB on a nearby rural background location was reported for the research site at Melpitz, Germany [19].

### 3.2. Spatiotemporal Variability of PAH Groups

**Figure 2:**
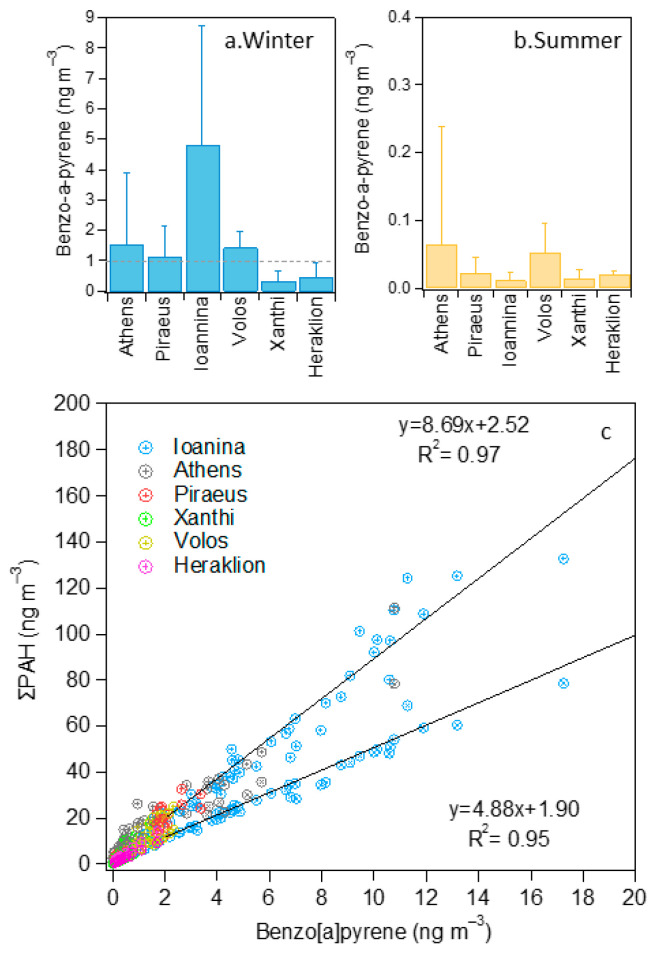
Seasonal variability of BaP mean concentration (**a**,**b**) at the six sites (dashed line for the EU target value) and correlation between the 16 EPA (high slope) and the 6 EU-proposed (low slope) PAHs with BaP (**c**).

The seasonal variability of the 16 EPA PAHs (Σ_16_PAHs) and BaP in the six cities is presented, including statistical testing, in Figure 2a,b and Figure S1 and Table 4. The winter/summer ratios of mean Σ_16_PAHs concentrations were 21, 7, 98, 16, 10 and 8 in Athens, Piraeus, Ioannina, Volos, Xanthi and Heraklion, respectively. For BaP, the corresponding ratios were 29, 60, 479, 29, 33 and 24. All seasonal differences were statistically significant (Table S1) and were related to the decreased anthropogenic emissions during summer in Greece, especially in the vacation month of August [26], but also to the increased volatilization of LMW PAH congeners. In addition, the enhanced atmospheric reactivity during the warm period led to PAH substitution or degradation [83] (this was particularly visible for BaP, the levels of which collapsed in summer across the country). High seasonal differences were observed for Σ_16_PAHs at the two urban background sites, Athens and especially Ioannina, as a result of strong RWB emissions in limited dispersion conditions that favor the appearance of intense pollution events (IPEs) in winter [18,30].

The lower ratio recorded in Piraeus (7 for Σ_16_PAHs), in spite of the very strong impact of winter RWB, which is comparable to the one at Athens—Thissio [31], indicates considerable PAH emissions from increased anthropogenic activity in the summer. This is related to the operation of the passenger port [31], as the sampling site is at a distance of approximately 150 m to the east of the port terminals (Figure S2a). The winter–summer gradient in Volos could also indicate increased emissions from the port area in summer (Figure S2c).

Heraklion also recorded a relatively low winter enhancement (ratio of 8 for Σ_16_PAHs), which should be related to the reduced RWB activity due to higher temperatures in winter but also to the local traffic source, which is associated with a more uniform emission profile throughout the year compared to residential heating. The rural background site in Xanthi also displayed a strong wintertime increase. This is expected since the site is affected mainly by regionally transported aerosol, which arrives at the site highly processed with respect to the source, and the majority of its original PAH content has been lost [84]. As discussed previously, an impact from RWB emissions in the city of Xanthi to the west is also possible (Figure S2b). Overall, the levels of both Σ_16_PAHs and BaP during summer were similar throughout Greece, with a Σ_16_PAHs range of 0.4–2.1 ng m^−3^ and a BaP range of 0.01–0.05 ng m^−3^, and the differences in the majority of the inter-site pairs were not statistically significant at the 99% confidence level (Table S2).

Figure S3 shows that during winter the PAH members with the highest concentrations at all sites were benzofluoranthenes (here presented as a BbjkF sum), followed by Chr in Ioannina, Xanthi and Volos, and by IP or BghiP in Athens, Piraeus and Heraklion. It can be observed that at the two sites in the Greater Athens Area (Athens and Piraeus), where a total of 3 million vehicles are circulating, the concentrations of high molecular weight (HMW) IP and BghiP were enhanced, indicating an important input from the road transport sector [85,86]. This was also the case for the traffic site in Heraklion. On the contrary, at the sites where in winter the RWB source dominates (especially in Ioannina), the Chr contribution to Σ_16_PAHs was stronger [87]. The third most abundant PAH members were IP, BghiP or BaP. It is noted that four of the five members (with the exception of BghiP), mentioned as the most abundant here, are found in the IARC list of carcinogenic agents.

The classification of PAHs by molecular weight is often used as indicative of their sources. For example, LMW PAHs (LMW: 128–178 gmol^−1^) and medium molecular weight PAHs (MMW: 202–228 gmol^−1^) have been used as indicators of heavy oil and diesel combustion emissions [88,89], while MMW and HMW (HMW: 252–300 gmol^−1^) PAH concentrations can be influenced by BB and road transport [90,91]. Also, it should be mentioned that the health impacts of PAHs are associated with their MW; for example, HMW member concentrations have been found to correlate with oxidative potential [92,93], while more volatile species have increased inhalation bio-accessibilities [94].

**Figure 3:**
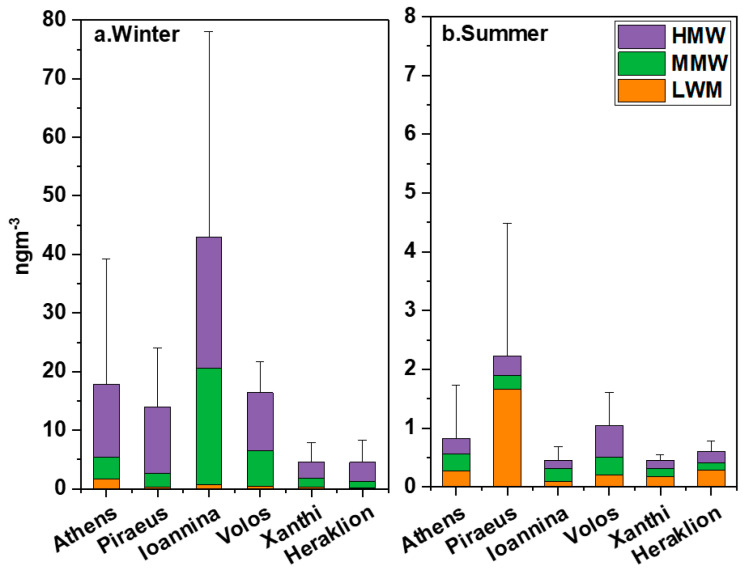
Seasonal variability of Σ_16_PAH concentrations by MW group in (**a**) winter and (**b**) summer in the six cities. Different colors represent the low- (LMW), medium- (MMW) and high- (HMW) molecular weight fractions.

Figure 3 presents the seasonal and spatial variation of mean PAH concentrations by MW group (the respective relative fractions in Σ_16_PAHs are shown in Figure S5). At all sites during winter, HMW PAHs exhibited the highest contributions (>50%), mainly demonstrating the importance of BB and car traffic emissions. Biomass burning is known to contribute strongly to HWM but also to MMW PAH members [95]. Especially, in Ioannina, it has been estimated that RWB can be responsible for approximately 92% of OC concentrations in winter [29] and is anticipated to be the major contributor to PAH concentrations as well. The only site that showed a substantial contribution of LMW PAHs during winter was Athens (~10%). At the Thissio site, which is a receptor site [23], a high contribution to Σ_16_PAHs (~33%) has been estimated by source apportionment analysis for a diesel/oil source (vehicles, central heating, shipping) enriched in LMW PAHs [18].

On the contrary, during summer, Piraeus was the only site exhibiting a majority contribution of LMW PAHs, which should be considered indicative of oil combustion in passenger ships navigating, calling at or hoteling in the port area [96,97]. Moreover, LMW PAH-emitting vehicular diesel emissions are an important contributor near the site in summer [98], with an increased number of heavy duty vehicles transporting goods to insular areas of Greece to accommodate tourist needs. The LMW PAH results in Piraeus during high temperature conditions, when a large part of LMW compounds volatilizes out of the particle phase [83,99], emphasize the strength of the source. Studies in other Mediterranean ports have presented similar summertime PAH levels and MW classification [75,100]. Even though LMW congeners such as Nap, Acy, Ace and Flu are volatile, they are regularly detected in the vast majority of PAH studies analyzing the PM_2.5_ phase and in comparable levels to the ones reported here [101,102]. In the present case, the highest concentrations of these LMW congeners were measured at the near-port site of Piraeus. It is characteristic that Tolis et al. in the port of Thessaloniki [103] reported six-fold higher levels of PM_2.5_-bound LMW members compared to Piraeus (e.g., mean annual Nap concentrations of 2.7 ng m^−3^).

In Ioannina, the mean summer concentrations of HMW and MMW species are more than two orders of magnitude lower compared to winter, due to the absence of the RWB source. Moreover, long-range transport might have had a lower effect in Ioannina as the area is shielded by high mountainous ranges. It is worth noting that the three sites that are more affected by combined port and traffic emissions (namely Piraeus, Volos and Heraklion) reported the highest summertime Σ_16_PAH levels, suggesting additional exposure risks for pedestrians, commuters and travelers [104].

For comparison reasons, the sum of the six PAHs proposed by the EU (Table S3) in its air quality directive is also included in Figure 2c. It can be observed that the average concentration difference when considering the 16 EPA and the 6 EU proposed PAHs is almost two-fold (Figure 2c and Figure S1). Furthermore, the excellent correlation seen between the Σ_16_PAHs (R^2^ = 0.97) and Σ_6_EU (R^2^ = 0.95) PAHs with BaP (Figure 2c) demonstrates that by determining the levels of BaP, a fair estimation of PAH sums can be provided for winter across Greece. It should be stressed that Σ_16_PAH levels presented some spatial variability during summer, in contrast to the sum of the six EU-suggested species - where this differentiation was not observed due to the non-inclusion of LMW PAHs that are increasingly present during the warm period mainly at the near-port sites. This within-area variability should be taken into consideration in the design of health studies aiming to characterize long-term PAH effects.

### 3.3. Diagnostic Ratios

Four diagnostic ratios (DR) were used, namely Ant/(Ant + Phe); Flt/(Flt + Pyr); BaA/(BaA + Chr); and IP/(IP + BghiP) (Figure S4). The first DR (Ant/(Ant + Phe)) involves LMW species and is useful for separation between petrogenic (<0.1) and pyrogenic (>0.1) origins [105,106]. The second DR (Flt/(Flt + Pyr)) includes MMW (202) PAHs and can suggest petrogenic (<0.4), non-solid fuel combustion (0.4–0.5) or biomass/coal burning (>0.5) sources [105,106]. BaA/(BaA + Chr) can indicate petroleum (<0.2), petroleum product combustion (0.2–0.35) and biomass/coal burning (>0.35) [34]. Finally, the ratio of the less volatile species (IP/(IP + BghiP)) is used in order to distinguish between petrogenic (<0.2), vehicular exhaust (0.2–0.5) and biomass/coal burning (>0.5) sources [107].

Τhe Ant/(Ant + Phe) ratio here indicated a predominantly pyrogenic origin in all cities, since its mean values were higher than 0.1. Only in Piraeus did the interquartile range (IQR) include the 0.1 cutoff, implying a potential influence of petrogenic PAHs deriving—from transport and storage activities of petroleum products in the port areas in Piraeus and the Elefsis bay [108]. Indicativly, 80% of ratio values below 0.1 in Piraeus were associated with prevailing winds from the port sectors (SW to NW). Both Athens and Piraeus presented wider IQRs compared to the other cities.

The Flt/(Flt + Pyr) ratio pointed to pyrogenic sources in all cities, registering mean values close to or above 0.4, again with the exception of Piraeus (although this discrepancy was marginal with a mean DR of 0.38). The mean DR in Ioannina appears to be lower than expected (<0.5) when considering the very large impact of RWB and the low temperatures that would ensure that the majority of the two MMW members were in the particle phase [83,109,110]. In addition, the ratio can be affected by the type of combustion appliance and the type of wood. In the region of Ioannina, hardwood is mostly used [29], and several studies have reported ratios smaller than 0.5 in hardwood burning emissions [111,112].

Conversely, the IP/(IP + BghiP) ratio, which depends on PAH members not being affected by volatilization for atmospheric chemical processing, verified the impact of RWB in Ioannina and Athens (mean values > 0.5), as it has already been hinted by the temporal variability characteristics. All other sites registered mean values within 0.45–0.5, indicating a mixed influence of wood and liquid fuel burning.

The BaA/(BaA + Chr) DR can also reveal the BB impact, as indicated by the >0.35 mean value in Ioannina, where in harsh winter conditions both species are found exclusively in the particle phase. Piraeus that was also affected by severe RWB episodes [31] had some very high daily DRs and a large IQR. Heraklion also had a high mean DR; however, this was affected by outlying values during festive days characterized by more intense BB emissions (e.g., Christmas).

**Figure 4:**
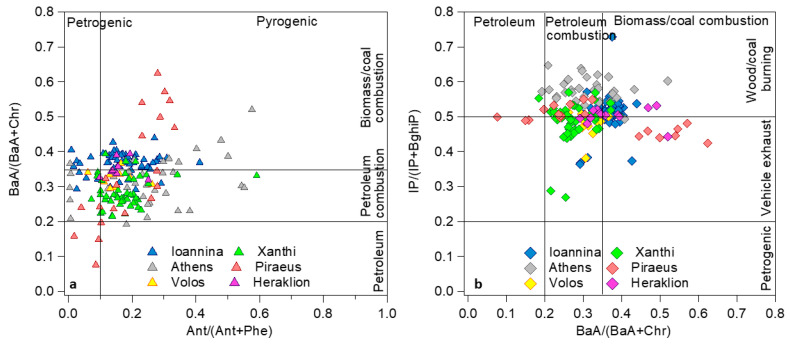
Diagnostic ratio (DR) cross-plot diagrams, (**a**) BaA/(BaA + Chr) to Ant/(Ant + Phe) and (**b**) IP/(IP + BghiP) to BaA/(BaA + Chr), based on PAHs concentrations measured in the six cities. DR ranges delineate the impacts of potential PAH sources.

Given that the ratios that involve volatile and reactive members [113] cannot fully represent the relative PAH abundances as they were during their emission, it is useful to examine DR cross-plots in order to reduce uncertainty. Figure 4 shows selected DR correlations for data-points from all sites combined during winter. The first cross-plot includes the ratios of BaA/(BaA + Chr) and Ant/(Ant + Phe) (Figure 4a) while the second presents IP/(IP + BghiP) vs. BaA/(BaA + Chr) (Figure 4b).

In Figure 4a, a petrogenic/petroleum origin is verified by both ratios only in the case of Piraeus. The majority of the data are associated with fuel burning, with a clear effect of BB being confirmed in Ioannina and also in Athens and Piraeus. There were, however, days in Ioannina that, despite high BaA/(BaA + Chr) ratios (>0.30), were classified as petrogenic by the Ant/(Ant + Phe) ratio. They all had Ant concentrations below the LOD and in their majority they were low-concentration days, which probably favored the almost complete degradation of Ant [30,114].

In the second cross-plot (Figure 4b), the impact of BB is confirmed for Ioannina [30,115], with the majority of points concentrated in the upper-right ninth of the grid. The important BB impact in Athens [18,116] was validated mainly by the HMW ratio, while the BaA/(BaA + Chr) was more balanced between vehicular and RWB emissions. Impacts from the use of both petroleum products and biomass can be deduced for Volos and Heraklion as well. In Piraeus, the large BaA/(BaA + Chr) ratios did not correspond to high IP/(IP + BghiP) values. Since the two HMW species are known to be enhanced by intense gasoline emissions, this behavior could indicate the coincidence of increased traffic and RWB in the holiday period [31]. In the rural background site, the impact of local BB emissions appears to be more limited, with the majority of the days linked to road fuel and oil combustion, although it should be noted that aerosols arrive at the Xanthi site highly processed and the typical ranges might not apply [117].

### 3.4. Associations of PAHs with Carbonaceous Compounds

PAH concentrations were also studied with respect to OC and EC measured concurrently at the same sites. The respective average OC and EC concentrations during winter and summer are listed in Table 5, along with their correlation coefficients with Σ_16_PAH concentrations.

Regarding the spatiotemporal variability of the relative OC and EC levels, at urban sites the mean OC/EC ratios were higher in winter than in summer, with differences being statistically significant at the 95% confidence level (Table S3), especially at the locations that were highly affected by RWB (Athens, Ioannina and Volos). The winter enhancement of OC average concentration at these sites was from 3-fold in Piraeus to 10-fold in Ioannina. While higher summer OC/EC in the past were reported in Greece due to the photochemical production of secondary organic aerosol (SOA), in the present conditions, it is typical for urban sites to record higher ratios in winter due to RWB emissions (especially in smoldering conditions) and their nighttime processing [77]. Xanthi had similar OC and EC levels in the two seasons, with a 47% higher OC/EC summer ratio being compatible with sampling of processed aerosol. OC levels in summer were within a 2-fold range (2.8–4.2 μg m^−3^) [26] but EC showed marked inter-site differences, resembling those observed for Σ_16_PAHs, with very low levels in Ioannina and Xanthi where high OC/EC ratios were recorded. In the remaining sites, the summer ratio of mean OC to mean EC was around 3 (including at the Heraklion traffic site), mostly indicating the impact of the road transport sector and a moderate local production of SOA (in Heraklion, traffic emissions that would lead to a lower OC/EC ratio of 1 to 2 are probably counterbalanced by the more enhanced photochemical production of SOA) [118,119]. The seasonal persistence of EC concentrations in Volos is also notable.

When comparing OC concentrations with both Σ_16_PAHs and BaP during winter, very strong Pearson correlation coefficients were observed (r = 0.91 and 0.89, respectively, for the pooled dataset across all sites). The strong associations were driven by co-emission, especially during nighttime when atmospheric dispersion is reduced in a shallow boundary layer and also by limited PAH volatilization. It has been shown that atmospheric reactivity in Greek cities is favored even in winter conditions; therefore, it is possible that processing mechanisms at a local level are similar between OC and PAHs. This could be supported by the fact that winter correlations of Σ_16_PAHs with inert EC are lower in comparison to OC, at least in the three cities where wintertime atmospheric chemistry has been verified in Greece [27,29,31].

The correlations of Σ_16_PAHs with OC were statistically significantly lower (*p*-value < 0.01) during summer, given the different degrees and pathways of processing that they are undergoing. This was also evident in the case of EC that is not transformed following co-emission with PAHs. A notable exception was the traffic site of Heraklion and also Piraeus that is also influenced by near-port traffic emissions.

**Figure 5:**
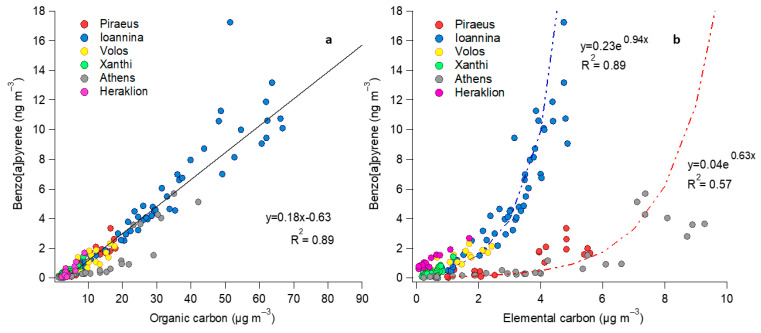
Correlations of BaP with OC (**a**) and EC (**b**), including winter data from the six sites.

Figure 5 displays the associations of BaP with OC and EC. It is apparent that since the correlation between OC and BaP during winter is excellent when considering all the urban sites (and the fact that the slopes of the OC vs. BaP linear associations remain stable among sites), OC might be used as a proxy for the estimation of BaP levels if needed, since the analysis for the determination of PAHs is more complex compared to OC. This observation could also be of use for urban-scale chemical transport models [120] to simulate PAH fields in winter conditions.

Figure 5b presents the association of BaP with EC across the cities in winter. In this case, the link was not linear, as with OC. Two patterns emerge, mostly influenced by the sites affected by winter smog events due to RWB. In Ioannina, the BaP/EC ratios during these events was lower than in the two sites in the area of Athens, where in spite of higher EC levels, BaP does not reach the high concentrations observed in Ioannina. The difference could be attributed either to differences in wood-burning type and conditions among the cities (it has been shown that while hardwood is mostly burned in Ioannina, in Athens, the biomass fuel mixture is more diversified), or to atmospheric conditions in Ioannina being more favorable for the conservation of BaP from atmospheric decay.

### 3.5. Aerosol Carcinogenic Risk

The BaP_eq_ values for all sites were calculated for summer and winter, taking into account the average concentrations and TEFs of the 16 EPA members (Table 6).

It is clear that the urban environment which exhibited the highest BaPeq values both during winter and overall was Ioannina. Athens, Piraeus and Volos experienced similar values in the range of 7–8 ng m^−3^, while the cities with the lowest values were Xanthi and Heraklion. Since these values are solely based on the 16 EPA components, they are expected to be significantly higher if other emerging PAHs are also taken into account [121]. At all sampling sites, benzofluoranthenes and dibenzo(ah)anthracene contributed the most to BaPeq (62–77%), while benzo(a)pyrene had smaller contributions (14–24%), highlighting the need to include more PAHs species to exposure studies for a more accurate estimation of risks.

Based on the estimated annual BaPeq values, the respective inhalation ILCR was calculated. As expected, the highest values were found for Ioannina, equal to 1.1 × 10^−5^ (OEHHA method) and 8.8 × 10^−4^ (WHO method). These values were well above the value of 10^−6^, which is considered a threshold above which carcinogenic risks become not acceptable, while the value calculated with the WHO method exceeded the 10^−4^ level, and have no alternatives for affordable residential heating other than biomass burning [122]. Athens, Piraeus and Volos were linked to similar ILCR values, in the order of 4.4–5.4 × 10^−6^ (OEHHA method) and 3.5–4.3 × 10^−4^ (WHO method). The lowest ILCR values were estimated for Xanthi, remaining, however, above the 10^−6^ lower acceptable threshold, while in Heraklion, values were 33% higher than Xanthi but not as high as in the other urban areas that are disproportionately impacted by RWB in winter. When comparing winter vs. summer BaPeq values, the risk in Athens was 44 times higher during winter, 35 times higher in Piraeus, a striking 78 times higher in Ioannina, 27 times higher in Volos, 24 times higher in Xanthi and merely 4 times higher in Heraklion. Comparing the values with other cities, the exposure levels were lower compared to Asian megacities like Xi’an (17 ng m^−3^) [123] and Taiyuan (28 ng m^−3^) [124]; however, the levels in Ioannina seemed to approach those extreme values. The other cities presented similar values to Zagreb, Croatia (4.5 ng m^−3^) [125], and Istanbul, Turkey (5.5 ng m^−3^) [126], but higher values than Gdynia, Poland (0.9 ng m^−3^) [127]; Győr, Hungary (1.4–2.2 ng m^−3^) [128]; southern Germany (2.7 ng m-3) [129]; Tuscany, Italy (0.1–0.8 ng m^−3^) [130]; and Santander, Spain (0.11–0.23 ng m^−3^) [131]. In conclusion, ILCR risks at all sites were above the minimum acceptable threshold, highlighting the significant impact of biomass burning on air quality and population exposure on a national level.

## 4. Conclusions

The levels of 16 EPA PAHs (Σ_16_PAHs) in six Greek cities were examined in two different periods (summer and winter) and were found to present substantial spatial (estimated annual means ranging between 2.5–21.7 ng m^−3^ among the cities) and temporal variability, driven by site type, local emissions and prevailing climatic conditions. Enhanced concentrations were observed during winter while in summer the levels remained uniformly low. Regarding estimated BaP mean annual levels, the EU target value is expected to be violated in Ioannina but also in other medium-sized cities in Northern Greece, which experience harsh winters and have no alternatives for affordable residential heating other than biomass burning.

Moreover, the separation of PAHs according to their molecular weight as well as diagnostic ratios of PAHs isomers and their cross-plots were used for the estimation of PAH sources. At all sites, the influence of local activities for residential heating was major during winter. During summer, PAH concentrations were mainly affected by fossil fuel emissions, linked to the road transport sector and to shipping in port areas (an observation that was verified for the first time by multi-site measurements in Greece), while the regional contribution was generally low. A direct correlation was observed for BaP and OC, while the correlation with EC presented patterns depending on the sampling site.

BaPeq estimates were used to evaluate the carcinogenic potency and estimate the incremental lifetime cancer risk from inhalation exposure to PAHs. The results indicate that there is a definite need to expand the range of PAHs (and their derivatives) that are presently considered in exposure and risk assessment studies, since there are members with recognized carcinogenic potential exceeding that of BaP and other IARC-listed species. The fact that five of the eight IARC-listed PAHs are in the HMW group indicates the large carcinogenic potential of the BB source, which needs to be regulated locally, if not nationally.

Among the six cities, the highest values were calculated in Ioannina, where the risks were found to be at alarming levels, with the severity of the issue being also intertwined with socioeconomic factors, given the low income in the region. This, on a national level, translates into substantial environmental injustice among regions and urban areas. Increased levels were recorded also in Athens, Piraeus and Volos. Despite the limitations of this study—mainly the unavailability of concurrent measurements at all sites, the use of different sampler types and the relatively small sample sizes in some cities—the results highlight the need to investigate PAH properties in more Greek cities and an urgent need for biomass burning regulations.

## Figures and Tables

**Table 1:** Details of sampling sites.

	Sampling Site	Type	Sampler Type	Model Type	Sampling Duration	Filter Samples
Athens	37.97° N, 23.72° E	Urban Background	Low volume (PM_2.5_)	3.1 PNS 15, Comde Derenda GmbH, Stahnsdorf, DE, USA	24 h	71
Piraeus	37.95° N, 23.64° E	Urban—Port	Low volume (PM_2.5_)	Derenda LVS-PNS-15; Comde-Derenda, Stahnsdorf, DE, USA	24 h	37
Ioannina	39.65° N, 20.85° E	Urban Background	High volume (PM_2.5_)	Digitel High Volume Aerosol Sampler DH77	24 h	81
Volos	39.36° N, 22.95° E	Urban—Port	High volume (PM_2.5_)	High volume automatic air sampler CAV-A/MSb (MCV S.A.)	24 h	29
Xanthi	41.15° N, 24.92° E	Rural Background	High volume (PM_2.5_)	Digitel High Volume Aerosol Sampler DH77	24 h	62
Heraklion	35.33° N, 25.14° E	Urban—Traffic	High volume (PM_10_)	Digitel High Volume Aerosol Sampler DH77	24 h	25

**Table 2:** Meteorological data by season for the six sampling sites.

	Winter			Summer		
	Temperature (°C)	Relative Humidity (%)	Wind Speed (m/s)	Temperature (°C)	Relative Humidity (%)	Wind Speed (m/s)
Athens	11.4 ± 2.1	64.9 ± 7.6	1.8 ± 0.7	28.2 ± 4.5	44.0 ± 13.6	1.9 ± 0.7
Piraeus	10.5 ± 2.2	68.2 ± 9.2	1.2 ± 1.0	28.8 ± 1.5	54.3 ± 9.8	1.3 ± 0.8
Ioannina	7.4 ± 2.4	70.5 ± 20.4	0.8 ± 0.5	26.8 ± 2.6	52.2 ± 9.3	0.7 ± 0.2
Volos	6.0 ± 1.5	68.2 ± 12.1	2.5 ± 1.1	27.5 ± 1.2	56.9 ± 4.3	2.3 ± 1.0
Xanthi	6.7 ± 3.1	73.6 ± 10.6	0.7 ± 1.0	25.9 ± 2.5	62.3 ± 10.6	0.8 ± 0.5
Heraklion	13.1 ± 2.6	77.3 ± 8.2	2.3 ± 1.2	26.0 ± 1.5	68.0 ± 8.6	2.1 ± 0.5

Statistical analysis for the determination of significant and non-significant differences between the seasons and sites was carried out using *t*-tests and analysis of variance (ANOVA).

**Table 3:** Information about the PAH species presented in this study.

Molecular Weight (g/mole)	Molecular Weight Group *	PAH Name	PAH Abbreviation	TEF **	IARC Classification	EU Air Quality Directive ***
**128**	LMW	Naphthalene	Nap	0.001	2B	x
**152**	LMW	Acenaphthylene	Acy	0.001		x
**154**	LMW	Acenaphthene	Ace	0.001	3	x
**166**	LMW	Fluorene	Flu	0.001	3	x
**178**	LMW	Phenanthrene	Phe	0.001	3	x
**178**	LMW	Anthracene	Ant	0.010	3	x
**202**	MMW	Fluoranthene	Flt	0.080	3	x
**202**	MMW	Pyrene	Pyr	0.001	3	x
**228**	MMW	Benzo(a)anthracene	BaA	0.200	2B	✓
**228**	MMW	Chrysene	Chr	0.100	2B	x
**252**	HMW	Benzo(b+j)fluoranthene	BbjF	0.800	2B	✓
**252**	HMW	Benzo(k)fluoranthene	BkF	0.200	2B	✓
**252**	HMW	Benzo(a)pyrene	BaP	1.000	1	✓
**276**	HMW	Indeno(123cd)pyrene	IP	0.100	2B	✓
**278**	HMW	Dibenzo(ah)anthracene	DBahA	10.000	2A	✓
**276**	HMW	Benzo(ghi)perylene	BghiP	0.009	3	✓

* LMW: low molecular weight; MMW: medium molecular weight; HMW: high molecular weight. ** Toxicity equivalent factor. *** Indication whether the congener is mentioned in the EU 2004/107/EC directive.

**Table 4:** Seasonal levels of the sum of 16 EPA PAHs (Σ_16_PAHs) and benzo[a]pyrene (BaP) at the six sites.

	Sum of Σ_16_PAHs (ng m^−3^)		Benzo[a]pyrene (ng m^−3^)	
	Winter	Summer	Winter	Summer
Athens	17.87 ± 21.37	0.87 ± 0.90	1.46 ± 2.33	0.05 ± 0.17
Piraeus	13.95 ± 10.1	2.15 ± 2.25	1.20 ± 0.99	0.02 ± 0.02
Ioannina	42.99 ± 35.08	0.44 ± 0.25	4.79 ± 3.92	0.01 ± 0.01
Volos	16.41 ± 5.24	1.05 ± 0.56	1.43 ± 0.53	0.05 ± 0.04
Xanthi	4.55 ± 3.37	0.45 ± 0.10	0.33 ± 0.26	0.01 ± 0.01
Heraklion	4.55 ± 3.72	0.61 ± 0.17	0.47 ± 0.47	0.02 ± 0.004

**Table 5:** Seasonal mean concentrations of organic and elemental carbon, and correlation coefficients for pairwise comparisons with mean concentrations for Σ_16_PAHs.

		OC (μg m^−3^)				EC (μg m^−3^)		
	Winter	r Σ_16_PAH	Summer	r Σ_16_PAH	Winter	r Σ_16_PAH	Summer	r Σ_16_PAH
Athens	16.5 ± 17.2	0.94	4.2 ± 1.4	0.47	3.9 ± 2.8	0.67	1.5 ± 0.8	0.16
Piraeus	9.8 ± 5.3	0.94	3.7 ± 0.8	0.25	3.2 ± 1.7	0.81	1.2 ± 0.5	0.63
Ioannina	28.2 ± 19.0	0.94	2.8 ± 1.0	0.02	2.8 ± 1.9	0.57	0.4 ± 0.2	0.21
Volos	11.9 ± 3.4	0.64	3.1 ± 0.7	0.30	1.5 ± 0.6	0.77	1.1 ± 0.6	0.32
Xanthi	3.8 ± 1.8	0.67	2.8 ± 0.7	0.13	0.4 ± 0.2	0.74	0.2 ± 0.1	0.18
Heraklion	4.4 ± 1.9	0.91	3.6 ± 0.7	0.31	1.3 ± 0.6	0.90	1.2 ± 1.1	0.73

**Table 6:** Seasonal variability of BaPeq, and ILCR estimates in the six cities.

	Athens	Piraeus	Ioannina	Volos	Xanthi	Heraklion
BaPeq * (ng m^−3^)						
Winter	9.59	8.34	19.98	7.77	2.14	2.66
Summer	0.22	0.24	0.26	0.29	0.09	0.56
Annual	4.91	4.29	10.12	4.03	1.12	1.62
ILCR ** (×10^−6^)						
OEHHA ***	5.4	4.7	11.1	4.4	1.2	1.8
WHO ****	427	373	880	350	97	141

* BaPeq: BaP-equivalent concentration for the mixture of the 16 EPA PAHs (Σ_16_PAHs). ** ILCR: incremental lifetime excess cancer risk. *** OEHHA: Office of Environmental Health Hazards Assessment of the California Environmental Protection Agency. **** WHO: Word Health Organization.

## Data Availability

Data are available upon request to the corresponding author.

## Supplementary Materials

The following supporting information can be downloaded at: https://www.mdpi.com/article/10.3390/toxics12040293/s1. Table S1: Statistical significance of seasonal differences in Σ_16_PAH concentrations measured at each site; Table S2: Statistical significance of inter-site differences in Σ_16_PAH concentrations during the warm season; Table S3: Statistical analysis between the OC/EC ratios during winter and summer at each site. Figure S1: Σ16-EPA PAHs and Σ6-EU PAHs mean concentrations at the six sampling sites (blue color for winter values; yellow for summer); Figure S2: Selected wind plots for Piraeus in summer (a), Xanthi in winter (b) and Volos in summer (c), associating wind direction with Σ_16_PAH concentration (ng m^−3^). The color scale indicates Σ_16_PAH levels and the radial axis shows their frequency of appearance by direction; Figure S3: PAH mean concentrations by member during winter (left) and summer (right) periods; Figure S4: Distribution of diagnostic ratio values calculated at the six sites; Figure S5: Seasonal variability of the relative contributions on LMW, MMW and HMW PAHs groups at the six sites.
