# Supplementary material for: Spatiotemporal Gradients of PAH Concentrations in Greek Cities and Associated Exposure Impacts
Source: Toxics. 2024 Apr 16;12(4):293. doi: 10.3390/toxics12040293 (PMC11055022; doi:10.3390/toxics12040293)
Supplement: Supplementary file 1 [file toxics-12-00293-s001.zip › toxics-2937171-supplementary.pdf]

Article

Spatiotemporal gradients of PAH concentrations in Greek cities and associated exposure impacts

I. Tsiodra[1], K. Tavernaraki[1,2], G. Grivas[1], C. Parinos[3], K. Papoutsidaki[2], D. Paraskevopoulou[1,2], E. Liakakou[1], A. Gogou[3], A. Bougiatioti[1*], E. Gerasopoulos[1], M. Kanakidou[2,4,5], and N. Mihalopoulos[1,2]

[1] Institute for Environmental Research and Sustainable Development, National Observatory of Athens, P. Penteli, 15236, Greece
[2] Environmental Chemical Processes Laboratory, Department of Chemistry, University of Crete, Heraklion, Crete
[3] Institute of Oceanography, Hellenic Centre for Marine Research, Anavyssos, 19013, Greece
[4] Center for Studies of Air Quality and Climate Change, Institute for Chemical Engineering Sciences, Foundation for Research and Technology Hellas, Patras, Greece
[5] Institute of Environmental Physics, University of Bremen, Bremen, Germany

* Correspondence: abougiat@noa.gr (A.B.)

Supplement
Tables:3
Pages: 5
Figures: 5

-

Table S1: Statistical significance of seasonal differences in Σ_{16}PAH concentrations measured at each site.
Table S2: Statistical significance of inter-site differences in Σ_{16}PAH concentrations during the warm season.
Table S3: Statistical analysis between the OC/EC ratios during winter and summer at each site.
Figure S1: Σ_{16}-EPA PAHs and Σ_6-EU PAHs mean concentrations at the six sampling sites (blue color for winter values; yellow for summer).
Figure S2: Selected wind plots for Piraeus in summer (a), Xanthi in winter (b) and Volos in summer (c), associating wind direction with Σ_{16}PAH concentration (ng m^{-3}). The color scale indicates Σ_{16}PAH levels and the radial axis shows their frequency of appearance by direction.
Figure S3: PAH mean concentrations by member during winter (left) and summer (right) periods.
Figure S4: Distribution of diagnostic ratio values calculated at the six sites.
Figure S5: Seasonal variability of the relative contributions on LMW, MMW and HMW PAHs groups at the six sites.

Table S1. Statistical significance of seasonal differences in Σ_{16}PAH concentrations measured at each site.

	P-value
**Athens**	7.68E-06
**Piraeus**	3.94E-05
**Ioannina**	3.93E-07
**Volos**	9.89E-12
**Xanthi**	1.63E-05
**Heraklion**	1.29E-03

Displayed *p*-values are derived by t-tests, and indicate statistical significance at the 99% confidence level in all cases.

Table S2. Statistical significance of inter-site differences in Σ_{16}PAH concentrations during the warm season.

	**Athens**	**Piraeus**	**Ioannina**	**Volos**	**Xanthi**	**Heraklion**
**Athens**		0.036	0.009	**0.400**	0.007	0.095
**Piraeus**	0.036		0.007	0.067	0.007	0.012
**Ioannina**	0.009	0.007		0.001	**0.953**	0.033
**Volos**	**0.400**	0.067	0.001		0.001	0.010
**Xanthi**	0.007	0.007	**0.953**	0.001		0.011
**Heraklion**	0.095	0.012	0.033	0.010	0.011	

Displayed *p*-values are derived by t-tests. It is apparent that mean concentrations between the sites are significantly different ($p<0.10$), apart from Xanthi-Ioannina, and similarities also exist between Volos-Athens. The results are also verified by analysis of variance (ANOVA) that indicates statistical significant variation ($p<0.01$) when all six sites are considered, but not when examining smaller subsets that include Ioannina and Xanthi ($p>0.05$).

Table S3. Statistical analysis between the OC/EC ratios during winter and summer at each site.

	OC/EC winter	*OC/EC summer*	*P-value*
**Athens**	4.06	3.01	7.56E-03
**Piraeus**	3.09	3.62	**3.13E-01**
**Ioannina**	9.79	6.99	2.19E-04
**Volos**	8.99	3.25	6.39E-07
**Xanthi**	8.99	21.17	6.25E-13
**Heraklion**	3.68	6.05	1.35E-02

It is apparent that average levels between winter and summer at all the sites are significantly different ($p<0.05$), apart from the site of Piraeus, due to the additional primary sources linked to traffic/shipping during summer.

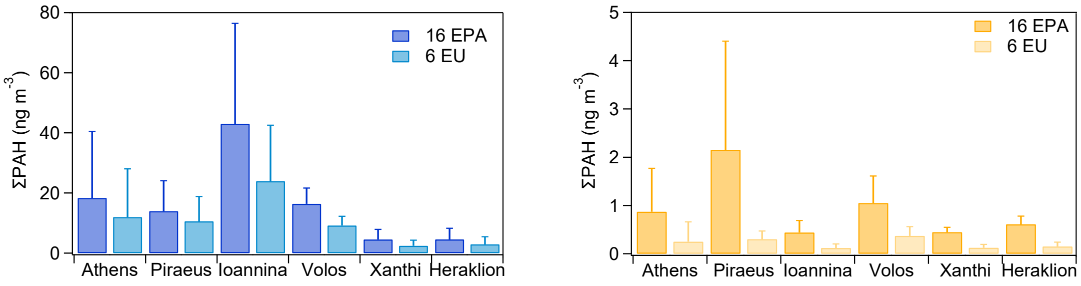

Figure S1. Σ_{16}-EPA PAHs and Σ_{6}-EU PAHs mean concentrations at the six sampling sites (blue color for winter values; yellow for summer).

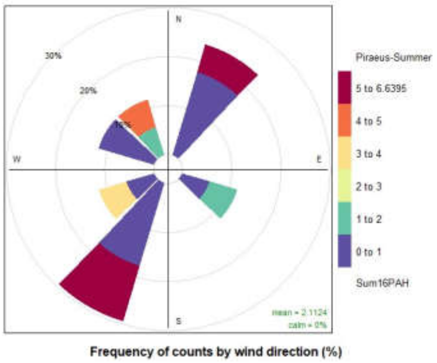

a

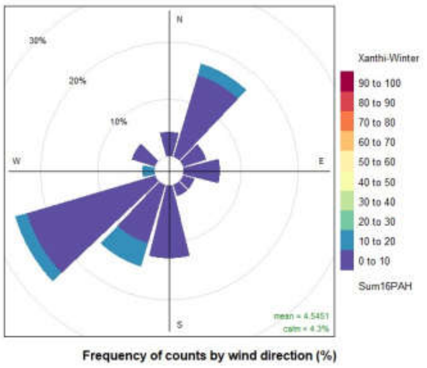

b

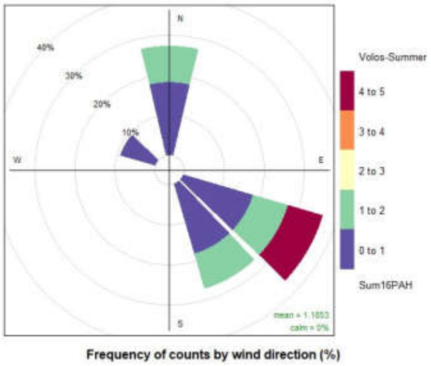

c

Figure S2. Selected wind plots for Piraeus in summer (a), Xanthi in winter (b) and Volos in summer (c), associating wind direction with Σ16PAH concentration (ng m^{-3}). The color scale indicates Σ_{16}PAH levels and the radial axis shows their frequency of appearance by direction.

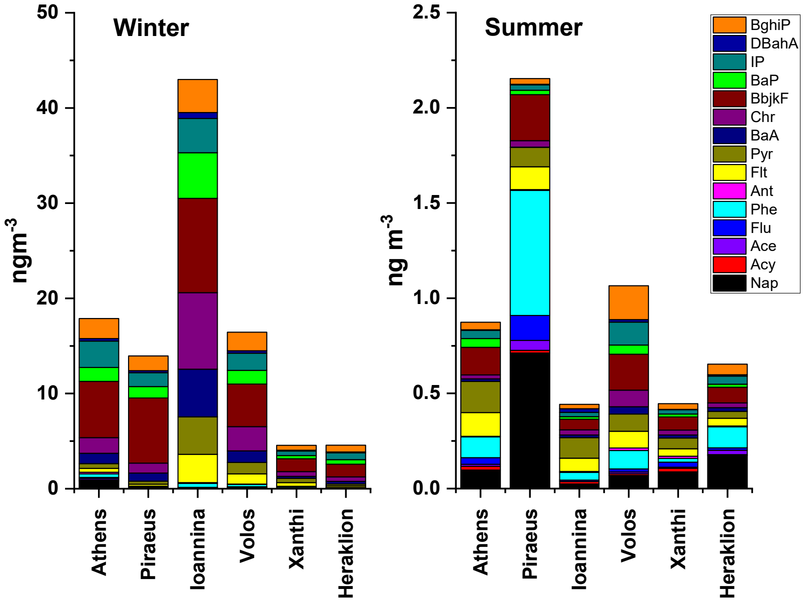

Figure S3. PAH mean concentrations by member during winter (left) and summer (right) periods.

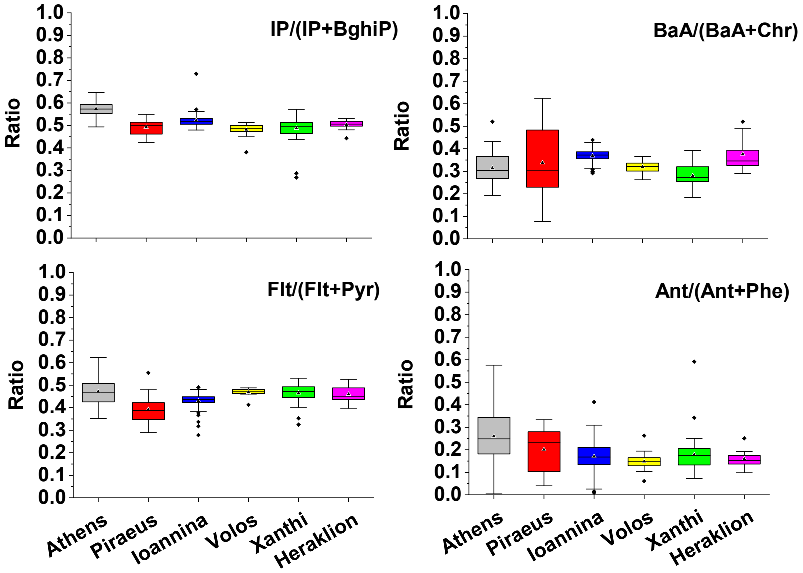

Figure S4. Distribution of diagnostic ratio values calculated at the six sites.

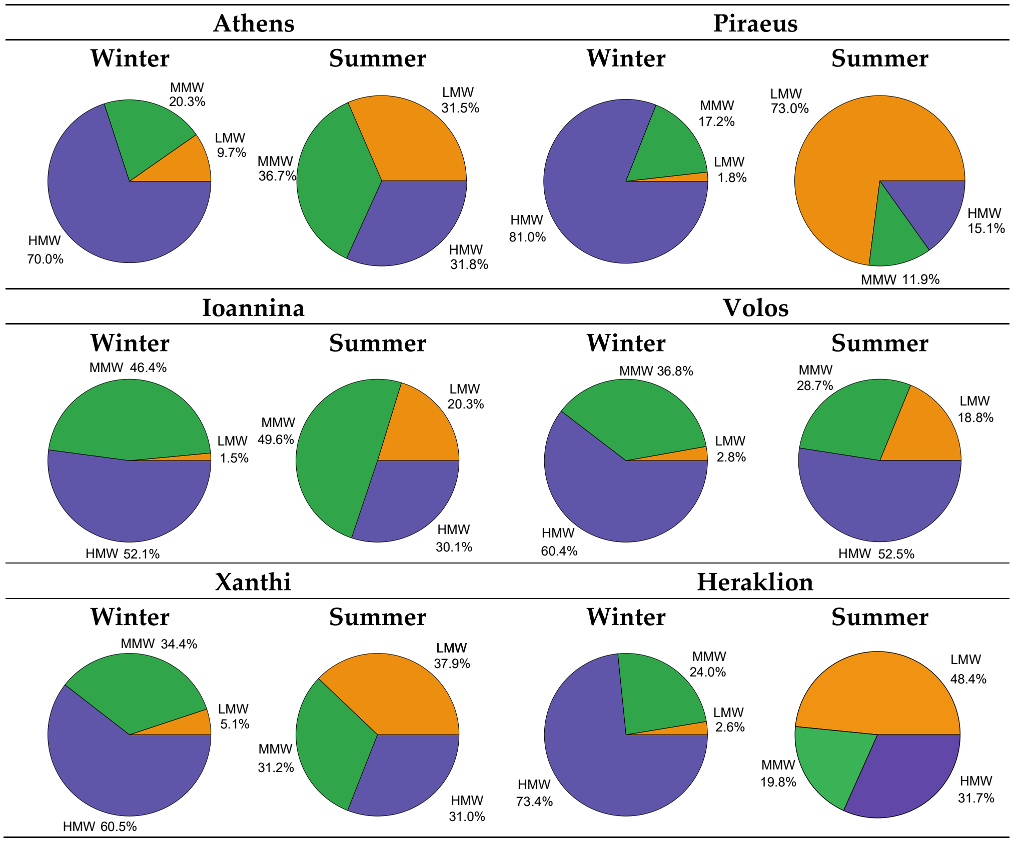

Figure S5. Seasonal variability of the relative contributions on LMW, MMW and HMW PAHs groups at the six sites.
